# New role of fat-free mass in cancer risk linked with genetic predisposition

**DOI:** 10.1038/s41598-024-54291-7

**Published:** 2024-03-27

**Authors:** Benjamin H. L. Harris, Matteo Di Giovannantonio, Ping Zhang, David A. Harris, Simon R. Lord, Naomi E. Allen, Tim S. Maughan, Richard J. Bryant, Adrian L. Harris, Gareth L. Bond, Francesca M. Buffa

**Affiliations:** 1 Department of Oncology, University of Oxford, Oxford, UK; 2 Wellcome Trust Centre for Human Genetics, University of Oxford, Oxford, UK; 3 St Anne’s College, University of Oxford, 56 Woodstock Rd, Oxford, UK; 4 Early Phase Clinical Trials Unit, Churchill Hospital, Oxford, UK; 5 Clinical Trial Service Unit and Epidemiological Studies Unit, Nuffield Department of Population Health, University of Oxford, Oxford, UK; 6 Nuffield Department of Surgical Sciences, University of Oxford, Oxford, UK; 7 Institute of Cancer & Genomics Sciences, University of Birmingham, Bimingham, UK; 8 Department of Computing Sciences, Bocconi University, Milan, Italy; 9 IFOM - Istituto Fondazione di Oncologia Molecolare ETS, Milan, Italy; 10 Cutrale Perioperative and Ageing Group, Imperial College London, London, UK

**Keywords:** Cancer, Breast cancer, Cancer epidemiology, Cancer genetics, Gastrointestinal cancer, Urological cancer

## Abstract

Cancer risk is associated with the widely debated measure body mass index (BMI). Fat mass and fat-free mass measurements from bioelectrical impedance may further clarify this association. The UK Biobank is a rare resource in which bioelectrical impedance and BMI data was collected on ~ 500,000 individuals. Using this dataset, a comprehensive analysis using regression, principal component and genome-wide genetic association, provided multiple levels of evidence that increasing whole body fat (WBFM) and fat-free mass (WBFFM) are both associated with increased post-menopausal breast cancer risk, and colorectal cancer risk in men. WBFM was inversely associated with prostate cancer. We also identified rs615029[T] and rs1485995[G] as associated in independent analyses with both PMBC (p = 1.56E–17 and 1.78E–11) and WBFFM (p = 2.88E–08 and 8.24E–12), highlighting splice variants of the intriguing long non-coding RNA CUPID1 (LINC01488) as a potential link between PMBC risk and fat-free mass.

## Introduction

Obesity is widespread across the globe^1,2^. Studies have identified high body mass index (BMI, weight in kilograms divided by the square of height in metres), a surrogate for adiposity, as a risk factor for cardiovascular disease, diabetes mellitus, chronic kidney disease and a number of cancer types^3–6^.

Although BMI has been described as a good proxy for assessing overall body fatness^6^, evidence from recent studies highlight its shortcomings^7,8^. Indeed, the well-described association of atrial fibrillation (AF) with obesity, as established mainly through BMI-based studies^9,10^, appears to be driven predominantly by lean rather than fat mass, challenging the role played by fat in the AF aetiology^11^. This finding opens up questions surrounding the use of BMI as a surrogate for obesity.

Currently, 13 cancer types are widely accepted to have sufficient evidence to be linked with “excess body fatness”, including post-menopausal breast cancer (PMBC), and malignancies of the bowel, endometrium, oesophagus, ovary, liver, gastric cardia, gallbladder, pancreas, kidney, meningioma, multiple myeloma and thyroid^6^. The majority of epidemiological studies have used BMI to identify links between cancer and obesity^12^, partly due to the ease of its measurement in large-scale population-based studies. Other researchers have studied changes in BMI or weight over time, and/or based their research on other indicators of adiposity, such as waist circumference and waist-hip ratio. A few studies have used more direct measures of fat mass, such as bioelectrical impedance, dual-energy X-ray absorptiometry or hydrodensitometry^13–16^.

Adipose and muscle tissue both have autocrine, paracrine and endocrine actions^17,18^, and these tissues have different physiological effects on whole body metabolism, inflammation, and insulin resistance^19,20^. Individuals with the same BMI can have very different body compositions of muscle and adipose tissue^21–23^. This concept has been used to explain the obesity/BMI paradox. This paradox refers to a number of studies demonstrating that overweight and early obese states are associated with improved survival in various cancers despite those with higher BMI being at increased risk of developing cancer^24–28^. This paradoxical observation has been attributed to individuals in overweight and early obese states having proportionally higher muscle mass contribution to BMI, but not having adiposity levels high enough to increase mortality^21^.

When considering cancer prevention, although there is a clear signal suggesting higher BMI individuals are at increased cancer risk for many cancer types, it is not evident where this signal originates within those individuals and/or whether this is the same across all tumour types. As in AF, it is possible that lean body mass (fat-free mass) is a risk factor for some cancer types, or perhaps signals from fat and lean body mass both culminate to increase cancer risk. It is crucial to resolve this issue, as it may change assessment of adiposity in future clinical trials, and refocus efforts to investigate the biological underpinnings of the observed links between obesity and cancer risk.

The UK Biobank presents an ideal opportunity to investigate the risk associated with BMI, fat mass and fat-free mass across a number of tumour types. This biobank is one of the world’s largest cohort studies of ~ 500,000 UK residents who volunteered to have their clinical, lifestyle, anthropometric and genetic data collected for health-related research. Notably, the vast majority of participants had bioelectrical impedance measurements taken at recruitment. This provides information on fat and lean body mass amounts and distribution. Further, participants have linked cancer incidence data from national health administrative datasets. Herein we report a study of three cancers in the UK Biobank, which were the most common in participants following anthropometric assessment (> 2000 cases), enabling robust phenotypic and genetic analyses. This includes two cancers that have been associated with high BMI (PMBC and colorectal cancer), and one cancer that has shown mixed results with BMI over a number of studies (prostate cancer). To date, most studies in the UK Biobank have focussed on measures of adiposity without assessing the relative contribution of lean body mass^29–31^. Here, we aim to address this gap in the literature by investigating what is the risk conferred by fat mass, fat-free mass and body mass index respectively in these three cancers.

Genetics play a crucial role in determining anthropometric measures and cancer risk, and help explain the mechanisms behind epidemiological associations. Over the past 10 years, genome-wide association studies (GWASs) have successfully identified loci that are associated with either cancer risk or anthropometric measures. While this has led to a greater understanding of the biology of these phenotypes, these studies focused on associations with only cancer types *or* anthropometric measures. The UK Biobank provides a unique opportunity to uncover the biological pathways linking some of these phenotypes. For instance, one may expect that if the link between fat mass and cancer risk is direct there will be loci associated with both traits (pleiotropy). We have previously demonstrated that single nucleotide polymorphisms (SNPs) affecting expression of key cancer genes associate with both cancer risk and anthropometric measures^32^. In this study, three regulatory SNPs for three important cancer genes, FANCA (rs1805007 C > T), MAP3K1 (rs889312, C > A) and TP53 (rs78378222, A > C) were found to associate with both anthropometric traits and cancer in a European population. The FANCA and MAP3K1 SNPs associated with height (standing height: rs1805007: beta =  − 0.02 ± 0.002, adjusted p = 9.20E − 15, MAP3K1: standing height beta =  − 0.02 ± 0.004, adjusted p = 6.11E − 14) and the TP53 SNP showed associations with 10 different anthropometric traits (rs78378222: multiple measures of height, fat-free mass and basal metabolic rate). Building on this work, we now search the entire genome for both fat and anthropometric-related genes linked to major malignancies.

## Methods

### The UK Biobank

The UK Biobank is a cohort of ~ 500,000 UK residents who volunteered to have their clinical, lifestyle, anthropometric and genetic data collected for research. Data for these analyses were collected at an assessment clinic at recruitment (2006–2010), and participants’ health was followed through linkage to electronic health records and centralised clinical registers, such as the Cancer register and Death register. Participants were aged between 40 and 69 years at recruitment. UK Biobank obtained informed consent from all participants. This study was conducted under the UK Biobank approved application (#43313, PI Francesca Buffa) and carried out in accordance with the Declaration of Helsinki. The National Research Ethics Service Committee approved all protocols.

### Anthropometric data

During the baseline assessment, participants had various anthropometric traits measured manually or by bioelectrical impedance. Standing height measurement was collected from participants using a Seca 240 cm height measure. Weight and bioimpedance data was measured using a Tanita BC418MA body composition analyser. Participants stood barefoot on the analyser and held the metal handles. This device produced measurements of weight, fat mass, and fat-free mass for the whole body and individual body segments (e.g. trunk fat mass), as detailed elsewhere^29^. The standing height measurement and weight was used in the calculation of BMI.

### Cancer data

Cancer occurrences were defined by the presence of a cancer international classification of diseases (ICD) code in the UK Cancer register or the UK Death register. To maximise the number of individual cancer cases, ICD9 and ICD10 codes were combined. The following ICD codes were used to identify patients with breast cancer (ICD-10: C50 (Malignant neoplasm of breast), D05 (Carcinoma in situ of breast), ICD-9: 174 (Malignant neoplasm of female breast), 2330 (Carcinoma in situ of breast)), colorectal cancer (ICD-10: C18 (Malignant neoplasm of colon), D010 (Carcinoma in situ of colon), 2303 (Carcinoma in situ of colon), C19 (Malignant neoplasm of rectosigmoid junction), C20 (Malignant neoplasm of rectum), D011 (Carcinoma in situ of rectosigmoid junction), D012 (Carcinoma in situ of rectum), 2304 (Carcinoma in situ of rectum), ICD-9: 153 (Malignant neoplasm of colon), 1540 (Malignant neoplasm of rectosigmoid junction), 1541 (Malignant neoplasm of rectum)) and prostate cancer (C61 (Malignant neoplasm of prostate), 185 (Malignant neoplasm of prostate), D075 (Carcinoma in situ of prostate)). Association analyses involving cancer data were repeated also without the in situ codes and results were similar.

### Epidemiological analysis

#### Analysis

Participants were removed from the analysis if there were missing data on anthropometric measurements, sex, age or had a recorded diagnosis of cancer before the assessment centre. This way only cancer incidence whilst on study was considered in the analyses, as the interest is in understanding the risk of developing a disease based on certain exposures or risk factors. Inclusion of prevalent cases can introduce bias, thus those with a prior diagnosis of cancer were excluded. For variables with missing data, to maximise statistical power we encoded all missing values in categorical and binary covariates with a new variable. Anthropometric measures were investigated as continuous and categorical variables. Baseline anthropometric measurements of the study participants were divided into quintiles. Correlations between anthropometric measures were calculated using Spearman’s rank correlation coefficient, which removes the need for reliance on a normal distribution of the underlying data.

Principal component analysis (PCA) was employed to study the relationship between different components of body composition and cancer risk. This approach overcomes the potential issues of overcorrection which can occur when intercorrelated parameters are used together in the same model. PCA is an adaptive exploratory method that serves as a descriptive tool that can be applied to a variety of data types. PCA needs no distributional assumptions^33^ and is commonly used to explore genomic datasets (e.g. microarray and RNA-Seq). Investigating associations with principal components themselves has yielded new biological insights^34^ but, as yet, PCA is rarely used in epidemiological studies particularly in this context. Here, PCA was carried out using Python (v.3.7). Principal components were created from 12 body composition variables related to fat mass and fat free mass: WBFM, WBFFM, trunk fat mass, trunk fat-free mass, Leg fat mass (right), Leg fat-free mass (right), Leg fat mass (left), Leg fat-free mass (left), Arm fat mass (right), Arm fat-free mass (right), Arm fat mass (left), Arm fat-free mass (left). PCA was carried out prior to the logistic regression and the principal components were also explored in logistic regression models.

Risk estimates for the association between the outcome and independent variables were calculated using reductionist models and multivariable-adjusted logistic regression models, adjusted for covariates. Odds ratios (ORs) and 95% confidence intervals (CIs) for the associations with anthropometric traits, notably Body Mass Index (BMI), Whole Body Fat-Free Mass (WBFFM) and Whole Body Fat Mass (WBFM) were estimated. In the multiply-adjusted models, cancer occurrence was examined using either BMI/WBFFM/WBFM accounting for only age of attendance to the assessment centre (5 year age group). Within the multivariable-adjusted models we accounted for well-accepted confounders selected a priori. PMBC models were adjusted for age of attendance to assessment centre (5 year age group), Townsend Deprivation Index, ethnicity, alcohol intake, smoking status, family history of breast cancer, diabetes mellitus, parity, oral contraceptive use, use of hormone replacement therapy, age at menarche, age at first full-term birth, physical activity (Metabolic equivalents in hours per week, METs) and age at menopause. Townsend Deprivation Index scores were derived from national census data about car ownership, household overcrowding, owner occupation, and unemployment aggregated for postcodes of residence. Higher Townsend scores equate to higher levels of socioeconomic deprivation. Data about household income were self-reported. Further, METs were used to quantify self-reported physical activity. This standard unit estimates the amount of energy expended while performing physical activities. One MET represents the amount of energy (calories) expended while sitting quietly. Multivariable prostate cancer models were adjusted for age of attendance to assessment centre (5 year age group), Townsend Deprivation Index, ethnicity, alcohol intake, smoking status, family history of prostate cancer, red meat intake (beef, lamb and mutton), fish intake (oily fish), diabetes mellitus, physical activity (METs), history of prostatitis, and regular use of aspirin, statins, saw palmetto, metformin, selenium supplements, Vitamin D supplements, Vitamin E supplements, testosterone supplements, use of anabolic steroids and 5 alpha-reductase inhibitors. Multivariable colorectal cancer models were adjusted for age of attendance to assessment centre (5 year age group), Townsend Deprivation Index, ethnicity, alcohol intake, smoking status, family history of bowel cancer, red meat intake (beef/lamb/mutton), physical activity (METs), diabetes mellitus, regular use of aspirin, statins, metformin, Vitamin D supplements in men. The same covariates were used in women plus menopausal status and use of hormone replacement therapy. Further information on the covariates is detailed in the UK Biobank showcase (https://biobank.ndph.ox.ac.uk/showcase/). Statistical tests for trend were calculated using the ordinal quintiles of each anthropometric measure entered into the model as a continuous variable. Statistical tests were all two-sided and a P-value < 0.05 was considered statistically significant. Logistic regression analyses were carried out using R (version 3.5.3).

### Genetic data

Blood samples were collected when participants were recruited, and DNA extracted. DNA was genotyped on either the Affymetrix UK BiLEVE Axiom array or the Affymetrix UK Biobank Axiom array (Santa Clara, CA, USA). Imputation was based upon a merged reference panel of ~ 90 million biallelic variants, from the 1000 Genomes Phase 3^35^ and the UK10K^36^ haplotype panels. Imputation was performed using IMPUTE2 as described^37,38^.

### Sample quality control and SNP quality check

In addition to the standard quality control, further quality control steps were carried out to ensure robustness of the analyses using data fields in the UK Biobank dataset. Individuals were excluded based on: a mismatched value between self-reported and genetic sex (data-field: 22,001 and 31), level of genotype missingness of > 0.05 (data-field: 22,005), genetic relatedness factor (kinship coefficient of > 0.0442, further details available at https://biobank.ctsu.ox.ac.uk/crystal/refer.cgi?id=531), sex chromosome aneuploidy (data-field 22,019), outliers for heterozygosity or missing rate (detailed in data-field: 22,027, outliers had heterozygosity > 0.1903). The European population was selected based on self-reported ethnicity (data-field: 21,000) by excluding non-white ethnic background. This left a study population of 379,453 suitable genotyped individuals.

SNP exclusions occured when at least one of the following conditions were met: Hardy–Weinberg equilibrium with p-value less than 1E-10, a minor allele frequency less than 0.0001, level of missingness more than 0.05 or an imputation score less than 0.8.

### Genomic association analyses

SNP-only based genetic association analysis of quantitative (anthropometric traits) and dichotomous (cancer occurrence) variables were carried out under an additive model using linear and logistic statistical framework, respectively, in PLINK v2.00a2LM. Age and genetic principal components (1–10 PCs) were used as covariates to control for hidden population structure in the genome-wide association study (GWAS). Sex was included as a covariate where appropriate, namely when testing anthropometric traits and colorectal cancer. All participants who had been diagnosed with cancer before the assessment centre were excluded from the analysis. Pleiotropic associations were selected following the steps below:

1. *Anthropometric associations:* SNPs significantly associated with BMI, WBFFM and WBFM were selected using a GWAS threshold-p of 5E-8.
2. *Haplotype calculation for anthropometric associated SNPs:* Haplotypes were calculated for the significant SNPs identified in step 1 (16,210 haplotypes) based on SNP pruning using PLINK v1.9 (R^2^ > 0.8 in Europeans of the 1000 Genomes Project phase 3 data). All UK Biobank SNPs without attributed rsID were excluded from the analysis.
3. *Search for cancer pleiotropic associations:* SNPs within haplotypes that significantly associated with anthropometric measures were assessed for association with cancers. Associations with post-menopausal breast, colorectal or prostate cancer were deemed significant if a SNP’s association-p was below a Bonferroni corrected threshold. The threshold was calculated by correcting 0.05 by the number of haplotypes calculated in step 2. The resultant threshold-p was 3.08E-6. Although sometimes deemed as conservative, the Bonferroni correction has several desirable properties^39^.

**Figure 1:**
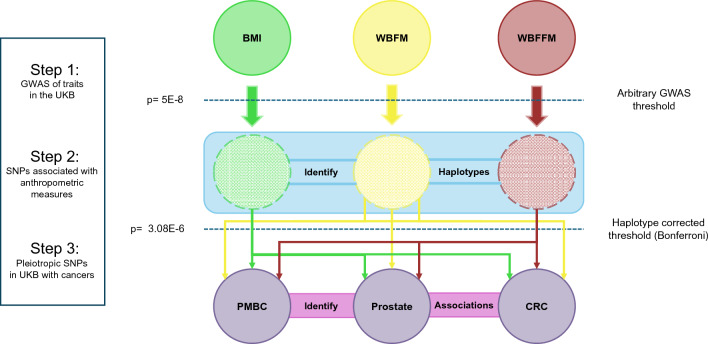
Workflow used to identify pleiotropic SNPs for anthropometric traits and cancer risk in the UK Biobank. In Step 1, SNP associations with BMI, WBFM and WBFFM were deemed significant if a SNP’s association-p was below an arbitrary GWAS threshold of 5E-8 (Bonferroni corrected). In Step 2, the resulting SNPs were then grouped into haplotypes (16,210). In Step 3, SNPs within the haplotypes were adjudged to have significant cancer associations according to a haplotype-adjusted Bonferroni corrected threshold (3.08E-6).

This workflow is summarised in Fig. 1.

### Expression quantitative trait loci

SNPs associated with differential gene expression were identified from expression quantitative trait loci (eQTL) databases: GTEX, NESDA/NTR and PancanQTL^40–42^.

## Results

### Association between cancer risk and measures of lean and fat body mass

Firstly, we selected common cancer types with at least 2000 cases from the UK Biobank cohorts to reduce false positives associations and increase the robustness of our findings. These identified PMBC, prostate and colorectal cancer as candidate cancer types. Using logistic regression models we investigated the relationship between the risk of these cancer types and each of BMI, WBFFM and WBFM. Quintiles were compared to the lowest (1st quintile) to give odds ratios for cancer risk. Reductionist and multivariable models yielded similar results and so the multivariable analysis is presented below. Number of cancer cases and cut points for each quintile are shown in Supplementary Tables S1 and S2. Using continuous age or categorial age did not materially affect the results. This analysis was complemented with PCA to investigate the relationships between different components of body composition and cancer.

### Risk of post-menopausal breast cancer is associated with BMI, WBFM and WBFFM

**Figure 2:**
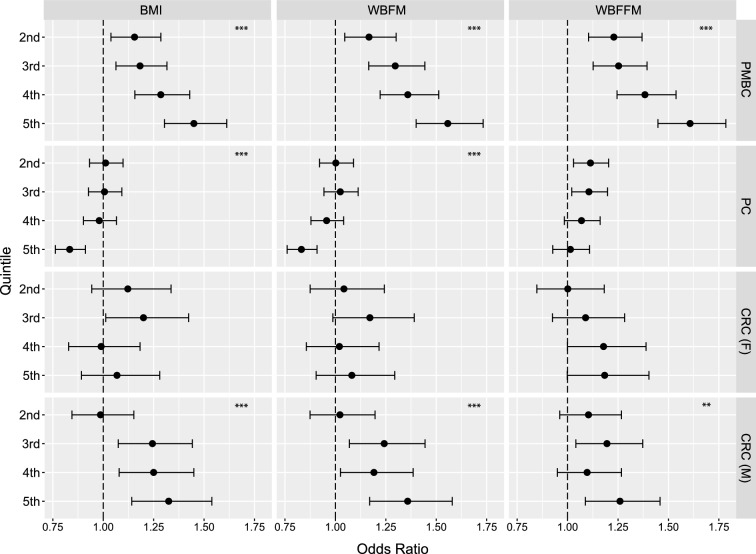
Association anthropometric traits with cancer risk. Odds ratio (95% CI) across quintiles of BMI, WBFM and WBFFM with post-menopausal breast, prostate and colorectal cancer (men and women are shown separately). Covariates for each model are detailed in the methods section. Quintile 1 is the reference category, with the 5th quintile denoting individuals in the highest fifth of each measurement. *BMI* body mass index, *WBFM* whole body fat mass, *WBFFM* whole body fat-free mass, *PMBC* post-menopausal breast cancer, *PC* prostate cancer, *CRC* (*F*) colorectal cancer in women, *CRC* (*M*) colorectal cancer in men. **p trend < 0.01, ***p trend < 0.001.

BMI, WBFFM and WBFM were each positively associated with an increased risk of PMBC to a similar extent (BMI quintile 5 = 1.45 (1.30–1.61) [BMI p trend = 2.86E–12], WBFM quintile 5 = 1.55 (1.40–1.73) [WBFM p trend = 1.19E–17], WBFFM quintile 5 = 1.60 (1.45–1.79) [WBFFM p trend = 2.52E–19], Fig. 2). BMI, WBFM and WBFFM were significantly correlated (p < 0.001) using Spearman’s rank correlation coefficient, with details shown in Supplementary Table S3a and illustrated in Supplementary Fig. S1. Notably, BMI and WBFM had an *r* value of 0.92, whereas BMI and WBFFM had an *r* value of 0.62.

PCA allowed identification of the main sources of signal affecting cancer risk. In PMBC, the first two PCs explained ~ 95% of variance (Supplementary Fig. S2a). Interestingly, the highest quintile of principal component 1 (PC1) was associated with an increased risk of PMBC compared to BMI, WBFM or WBFFM alone (Supplementary Fig. S2b). Most of the contribution of PC1 is given by whole body fat mass, trunk fat mass and whole body fat-free mass (Supplementary Fig. S2c).

### Risk of prostate cancer is inversely related with measures of body fat

Compared to the lowest category of BMI and WBFM, men in the highest category had a lower risk of prostate cancer (BMI quintile 5 = 0.83 (0.76–0.91) [BMI p trend = 1.82E–04], WBFM quintile 5 = 0.83 (0.76–0.91) [WBFM p trend = 4.27E–05], Fig. 2). WBFM and BMI had a slightly less positive coefficient than in the PMBC, *r* = 0.89 (Supplementary Table S3b), and the correlation coefficient between WBFM and WBFFM was 0.53 (p < 0.001), Supplementary Fig. S3).

The first two PCs explained > 93% of variance (Supplementary Fig. S4a). Looking at PC1, the 5th quintile just reached significance as being associated with a reduced risk, but the confidence intervals around the OR almost included 1 (Supplementary Fig. S4b). PC2 (~ 18% of explained variance) showed differential directional relationships with WBFM and WBFFM. Interestingly, the highest quintile in PC2 was associated with similarly reduced risk as the highest quintile of whole body fat mass. Most of the contribution of the PC2 was given by WBFM (Supplementary Fig. S4c).

### Risk of colorectal cancer is associated with BMI, WBFM and WBFFM in males but not females

Colorectal cancer was investigated in a sex-specific analysis, as previous work has demonstrated different associations in men and women^43^. In women, all confidence intervals included 1 or were very close to including 1 for all anthropometric measures (Fig. 2). As seen in the post-menopausal cohort correlation analysis, BMI and WBFM were strongly correlated (*r* value of 0.93) while BMI and WBFFM were less so (*r* value of 0.63) (Supplementary Table S3c and Supplementary Fig. S5).

The first two PCs in colorectal cancer in women explained ~ 95% of variance (Supplementary Fig. S6a). Different quintiles of PC1 did not appear to carry significantly different risks of colorectal cancer. The last quintile of PC2 was associated with a small increased risk for colorectal cancer, which is difficult to interpret alone (Supplementary Fig. S6b). Most of the contribution of the PC1 in women was given by WBFM and WBFFM, with WBFM showing a larger loading value (0.809, Supplementary Fig. S6c) compared to its contribution to PCA1 in men (0.641).

When considering colorectal cancer in men, BMI and WBFM were positively associated with colorectal cancer risk (BMI quintile 5 = 1.32 (1.14–1.54) [p trend = 2.95E–06], WBFM quintile 5 = 1.36 (1.17–1.58) [WBFM p trend = 7.41E–06], Fig. 2). The trend of WBFFM was also significant (WBFFM quintile 5 = 1.26 (1.09–1.46) [WBFFM p trend = 6.28E–03]). Correlation coefficients were very similar to the prostate cancer analysis cohort (Table S1d and Supplementary Fig. S7).

Here, the first two PCs explained ~ 92% of variance (Supplementary Fig. S8a/b). Increasing quintiles of PC1 were associated with increased risk. Most of the contribution of the PC1 was given by WBFM and WBFFM, perhaps pointing to both having some element of risk (Supplementary Fig. S8c).

### Identification of pleiotropic SNPs through genomic association analyses

Having demonstrated that: (i) increasing BMI, WBFM and WBFFM were associated with increased PMBC risk, (ii) increasing WBFM was associated with reduced prostate cancer risk and (iii) increasing WBFFM and WBFM were associated with colorectal cancer in men, we hypothesised that genetic loci would show similar pleiotropic associations. To test this hypothesis, we explored the genetic data in the UK Biobank and performed GWASs on BMI, WBFFM, WBFM, PMBC, prostate and colorectal cancer.

**Figure 3:**
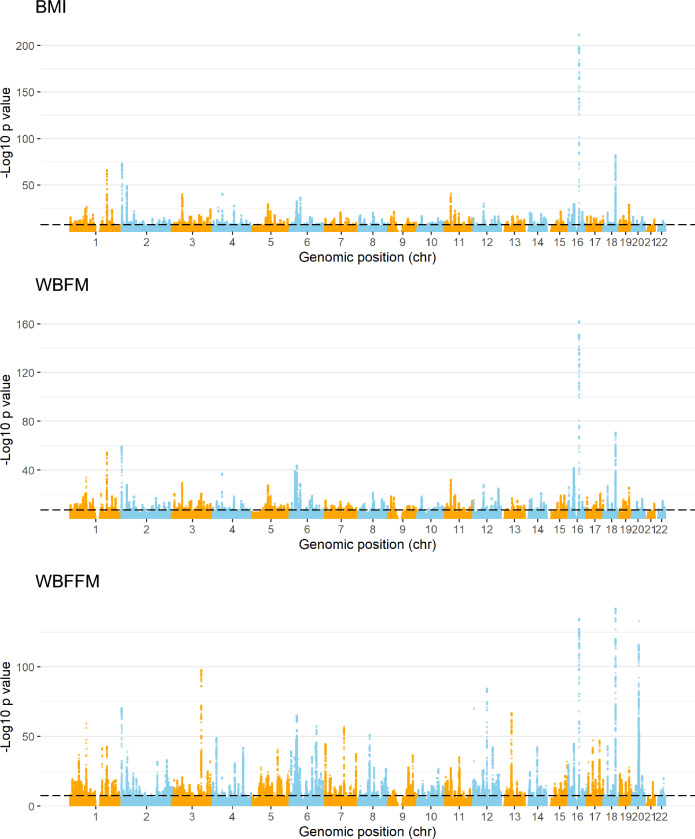
SNPs associated with BMI, WBFFM and WBFM in the UK Biobank. Genetic association analyses were carried out using the UK Biobank genomic data through PLINK. Results are displayed here as Manhattan plots. Horizontal axes show chromosome loci and vertical axes show – log_10_(p) value of association. A high vertical distance indicates a more significant association. The broken horizontal line represents an arbitrary GWAS threshold of 5E-8 which was used to denote a significant association.

This revealed 46,825 SNPs significantly associating with BMI (lowest p: 2.17E–213, beta = 0.076, rs11642015[T]), 85,378 SNPs significantly associating with WBFFM (lowest p: 5.10E–143, beta = 0.040, rs6567160[C]) and 41,053 SNPs significantly associating with WBFM (lowest p: 2.88E-163, beta = 0.065, rs11642015[T]) (Manhattan plots in Fig. 3 and further details in Supplementary Tables S4–6).

**Figure 4:**
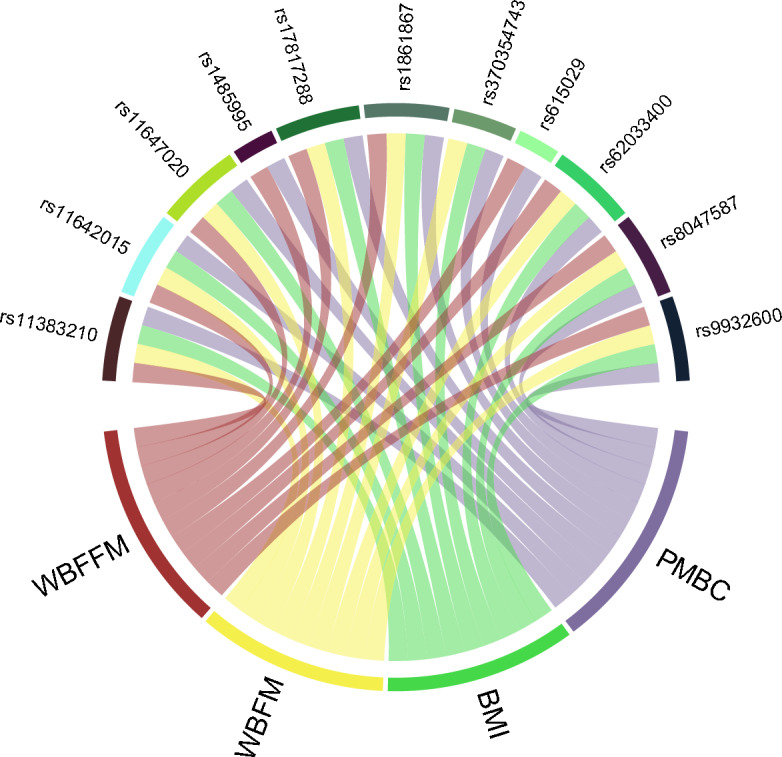
Pleiotropic haplotypes associated with both post-menopausal breast cancer and anthropometric measures. Genetic association analysis of quantitative (anthropometric measures) and dichotomous (cancer occurrence) variables were carried out under an additive model in PLINK using the UK Biobank genetic data. Age and genetic principal components (1–10 PCs) were used as covariates to control for hidden population structure in the GWAS of all phenotypes investigated. Pleiotropic lead SNPs that have significant associations with both post-menopausal breast cancer (PMBC) and anthropometric measures (association p < 3.08E-6 on both phenotypes) are shown. *WBFFM* whole body fat-free mass, *WBFM* whole body fat mass, *BMI* body mass index.

In the PMBC analysis, 11 haplotypes were identified to be associated with both PMBC and anthropometric traits (Fig. 4, Supplementary Table S7). Three of these haplotypes were positively associated with PMBC risk and anthropometric measures, with the same directions as our epidemiological models. One of these three haplotypes was associated with increased BMI and WBFM, and increased risk of PMBC. The solitary SNP within this haplotype, rs370354743[TA], a 3’UTR Variant in SKI/DACH Domain-Containing Protein 1 (SKIDA1), was associated with PMBC (p = 4.68E–07, OR: 1.09), BMI (p = 4.86E–13, beta = 0.02) and WBFM (p = 1.61E–12, beta = 0.02). This SNP has not been described to associate with these traits in the GWAS catalog. Further, it does not appear to influence gene expression when interrogating the eQTL databases.

The other two haplotypes were positively associated with WBFFM and increased PMBC risk. The lead SNP in one haplotype, rs1485995[G], was associated with PMBC (p = 1.78E–11, OR: 1.11) and WBFFM (p = 8.24E–12, beta = 0.01) while the lead SNP in the other haplotype, rs615029[T], was associated with PMBC (p = 1.56E–17,OR 1.19) and WBFFM (p = 2.88E–08, beta = 0.01). We were able to validate a breast cancer association of a SNP in this haplotype, rs537626[C], previously identified in a case–control study of 15,170 individuals with 6993 cases (p = 1.8E–15, OR: 1.29) by other workers^44^. Interrogation of eQTL databases showed SNPs in these haplotypes are not recorded to be associated with changes in gene expression. This is expected as rs537626[C] is a non-coding transcript exon variant related to long intergenic non-protein coding RNA 1488 (LINC01488) otherwise known as CUPID1 (*CCND1 Upstream intergenic DNA Repair 1, non-coding*). This would not be covered by standard eQTL analyses.

We identified one haplotype where some SNPs associated positively and some negatively with PMBC, BMI, WBFM and WBFFM within the same haplotype. The SNP with the strongest association with BMI, WBFFM and WBFM, rs17817288[G], is an intron variant mapped to the fat mass and obesity-associated (FTO) gene. SNPs in this haplotype have not been seen to associate with cancer risk or anthropometric traits in the GWAS catalog. However, various other FTO intron variants have been associated with these phenotypes, including rs7193144[T] with BMI^45^ and rs11075995[A] with breast cancer^46^. The other seven haplotypes are found in a similar chromosomal region to the FTO haplotype SNPs and are associated with anthropometric measures and PMBC risk in opposing directions (Supplementary Table S7).

In the prostate cancer analysis, 17 haplotypes were identified that associated with anthropometric measures. All 17 were associated with WBFFM, two of these also associated with WBFM and one with BMI (Supplementary Table S8). However, these haplotypes showed discordant directional associations between prostate cancer and anthropometric measures to our epidemiological models. The pleiotropic SNP that was closest to mirroring the associations was rs377763[A], which was negatively associated with prostate cancer (p = 7.58E–07, odds ratio = 0.90), and positively associated with BMI (p = 9.34E–10, beta = 0.02), WBFM (p = 9.79E–12, beta = 0.02), and WBFFM (p = 3.30E–33, beta = 0.02). This SNP is an intergenic variant close to notch receptor 4 (NOTCH4) and is an eQTL for various genes in multiple tissues in the GTEX database, including NOTCH4, Cytochrome P450 Family 21 Subfamily A Member 2 (CYP21A2, which codes for 21-hydroxylase enzyme) and Complement C4A (C4A).

No SNPs exhibited pleiotropic associations with colorectal cancer and the anthropometric measures tested. Further, the lists of SNPs associated with colorectal cancer, prostate cancer and PMBC are found in Supplementary Tables S9–11.

## Discussion

In this study we aim to address an important knowledge gap on the link between the different components of body mass and cancer risk. We describe different associations of BMI, WBFFM and WBFM within three cancer types in the UK Biobank using comprehensive multivariable models. We demonstrate that pleiotropy exists on a genome-wide scale with SNPs being simultaneously associated with differential cancer risk and anthropometric measures in the same large cohort. Importantly, we identify loci exhibiting pleiotropic associations that may shed light on potential mechanisms behind our epidemiological observations.

In our initial analyses, we show that the associations between anthropometric traits and cancer risk are specific to the cancer types. For example, we show that increasing BMI, WBFFM and WBFM are each associated with increasing PMBC risk and principal component analysis shows that both WBFFM and WBFM contribute to PMBC risk. In contrast, men with higher BMI and WBFM had a lower risk of prostate cancer, with the relationship of WBFFM being less clear. For colorectal cancer, there was no clear relationship with WBFM or WBFFM in women, whereas in men, the highest quintiles of WBFM and WBFFM are both significantly associated with increased risk with principal component analysis suggesting both factors may contribute to this increased risk.

Various hypotheses have been proposed to explain the increased risk for PMBC and male colorectal cancer with increased fat-mass (WBFM), as described here. These include adipocyte hypertrophy and death leading to systemic chronic, subclinical inflammation of adipose tissue^47^. Also, leptin and oestrogen from adipocytes and increased circulating insulin caused by insulin resistance may act as growth factors^48–50^. Further discussions on mechanisms relating cancer and obesity is found in the following references^51,52^. Additionally, we comprehensively show a link between WBFFM and PMBC risk, as has been shown previously in smaller cohorts^16,53^. The causal mechanism is unknown, but it is possible that insulin-like growth factor 1 (IGF-1) might be involved, as this has anabolic effects and has been associated with cancer risk in large-scale observational studies^54^.

The reported observation that those with higher BMI have a reduced overall risk of prostate cancer, is consistent with some studies^55,56^. However, obesity (as defined by BMI) has been associated with more aggressive prostate cancer in previous studies, with the suggestion this might be due to the development of a low testosterone/high estradiol environment^57^. Opposing effects on cancer risk and progression are suggestive of different drivers, and including information on stage and grade of prostate cancer to the UK Biobank resource would enable further research into the relationship between more direct measures of adiposity and disease aggressiveness.

In the current study, we used GWAS to identify genetic loci with pleiotropic effects on both anthropometric traits (notably WBFM and WBFFM) and cancer. Our approach revealed pleiotropic haplotypes (co-inherited SNPs) involving prostate and PMBC. The pleiotropic SNP rs377763[A] which was negatively associated with prostate cancer (p = 7.58E–07, OR 0.90) and positively associated with BMI (p = 9.34E–10, beta = 0.02), WBFM (p = 9.79E–12, beta = 0.02), and WBFFM (p = 3.30E–33, beta = 0.02). This eQTL influences NOTCH4 expression in a range of tissues, which may affect downstream pathways affecting proliferation, including Wnt1/ β-catenin signalling^58^.

SNPs within one haplotype associated with both PMBC (e.g. rs615029G > T, adjusted-p = 1.56E–17, OR 1.19) and WBFFM (e.g. rs615029G > T, adjusted-p = 2.88E–08, beta = 0.01) providing a potential mechanistic link. Further, one SNP within the same haplotype, rs537626[C], has previously been found to associate with breast cancer in a different cohort^44^. This polymorphism (rs537626[C]) is a non-coding transcript exon variant related to a long non-coding RNA (lncRNA), LINC01488/CUPID1. LncRNAs are present in low copy numbers and do not code for polypeptides, although they are extensively processed by splicing (considerably more than mRNA itself) and 3’polyadenylated^59^. Indeed, nearly every non-coding exon is subject to alternative splicing^60^. rs537626[C], and other SNPs in this haplotype, may well alter the function of this lncRNA.

CUPID1 is induced after oestrogen stimulation, and most likely affects pathway choice for DNA repair^61,62^. Silencing of CUPID1 inhibits RAD51 recruitment to double-strand breaks, and appears to decrease high-fidelity homologous recombination through a defect in end resection. This results in the more mutagenic non-homologous end-joining (NHEJ) becoming the dominant DNA-repair pathway^61^. Alternatively, homologous recombination might be reduced due to its effects on the cell cycle. CUPID1 is transcribed from DNA within the promoter region of cyclin D, acting as a brake on cyclin D production, via effects on histone acetylation. This slows cell proliferation, particularly in the S phase where homologous recombination is favoured. In its absence, the cells more rapidly progress through S phase and NHEJ becomes the preferred repair pathway. This SNP may thus influence breast cancer risk through the creation of new, and persistence of existing, mutations. CUPID1 also decreases stability of cyclin E, decreasing the production of pro-metastatic agents such as vimentin (via increasing the level of inhibitor miRNA species miR124-3p and miR138-5p) and MMP9^63^. The link between increased fat-free mass and rs537626[C] (and other SNPs within its haplotype) is less clear as we were unable to confirm alterations in gene expression through interrogation of eQTL databases. This is an area for further study. As is looking for interactions between SNPs/haplotypes and anthropometric measures and cancer risk using other machine learning methods.

This study has some relevant limitations. Firstly, limited information is currently available on aspects of the cancers in the UK Biobank. At the time of writing, no information on stage, grade, histological type and treatment status was available. Further, prostate specific antigen (PSA) testing was not carried out as standard on all participants. Some cancer types can have a lengthy aetiology, so it is possible some individuals had undiagnosed malignancies prior to the assessment centre. This may lead to a protopathic bias which cannot be definitively excluded in this study. It should be noted that the accuracy of bioelectrical impedance measurements (relative to other techniques) is debated in some circles, as these measurements can be affected by factors such as hydration status, impact of exercise and fluid retention (even to the level of the menstrual cycle)^64,65^. The true impact of these factors is unknown in this dataset. In addition, as other large datasets with WBFM and WBFM become available, it will be interesting to investigate and attempt to validate interactions between haplotypes and anthropometric measures and cancer risk using machine learning approaches.

Our current study is novel and the first in integrating epidemiological clinico-demographic and genome-wide approaches and, using bioelectrical impedance to study relationship between BMI, WBFM and WBFFM and cancer risk. We demonstrate that these anthropometric measures carry different risk profiles in common cancer types, and this underlines the importance of not investigating these elements in isolation in future studies. We provide evidence that increasing WBFFM is associated with increased PMBC risk and identify splice variants of CUPID1 as a potential link between these two clinical phenotypes.

### Supplementary Information

Supplementary Figure 1.

Supplementary Figure 2.

Supplementary Figure 3.

Supplementary Figure 4.

Supplementary Figure 5.

Supplementary Figure 6.

Supplementary Figure 7.

Supplementary Figure 8.

Supplementary Legends.

Supplementary Table 1.

Supplementary Table 2.

Supplementary Table 3.

Supplementary Tables.

Supplementary Tables.

## Data Availability

The datasets generated and/or analysed during the current study are not publicly available because the data for this study was made available under an application to the UK Biobank consortium. We are not at liberty to make their data public. Access is controlled and researchers can apply for this data directly from the UK Biobank consortium. Data are available through an application to the UK Biobank (https://www.ukbiobank.ac.uk/) and also are available from the corresponding author with blessing from the UK Biobank consortium on request.
