# Supplementary figures and images for: New role of fat-free mass in cancer risk linked with genetic predisposition
Source: Sci Rep. 2024 Mar 27;14:7270. doi: 10.1038/s41598-024-54291-7 (PMC10973462; doi:10.1038/s41598-024-54291-7)

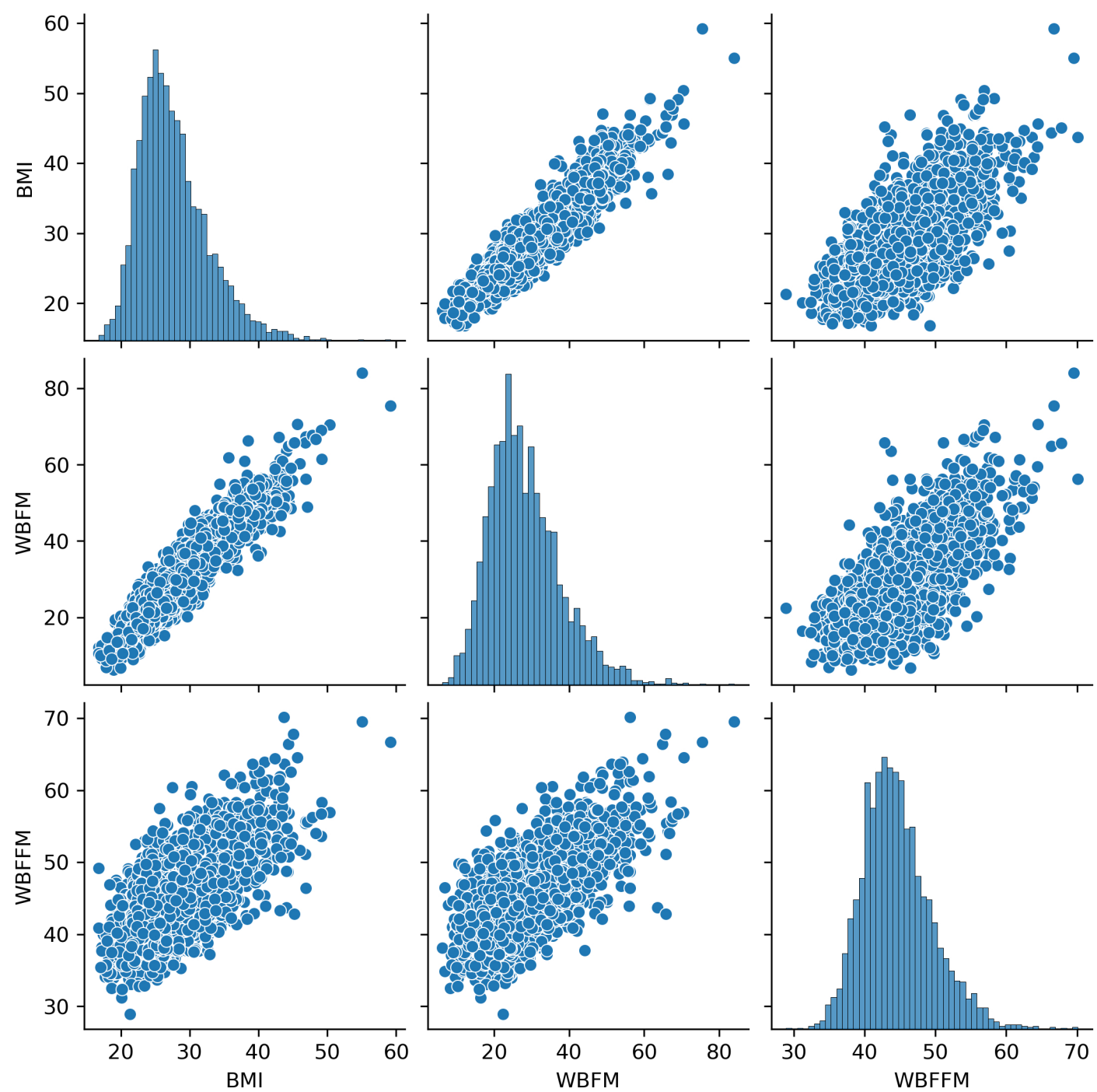

Supplement: Supplementary file 1 — Supplementary Figure 1. [file 41598_2024_54291_MOESM1_ESM.pdf]

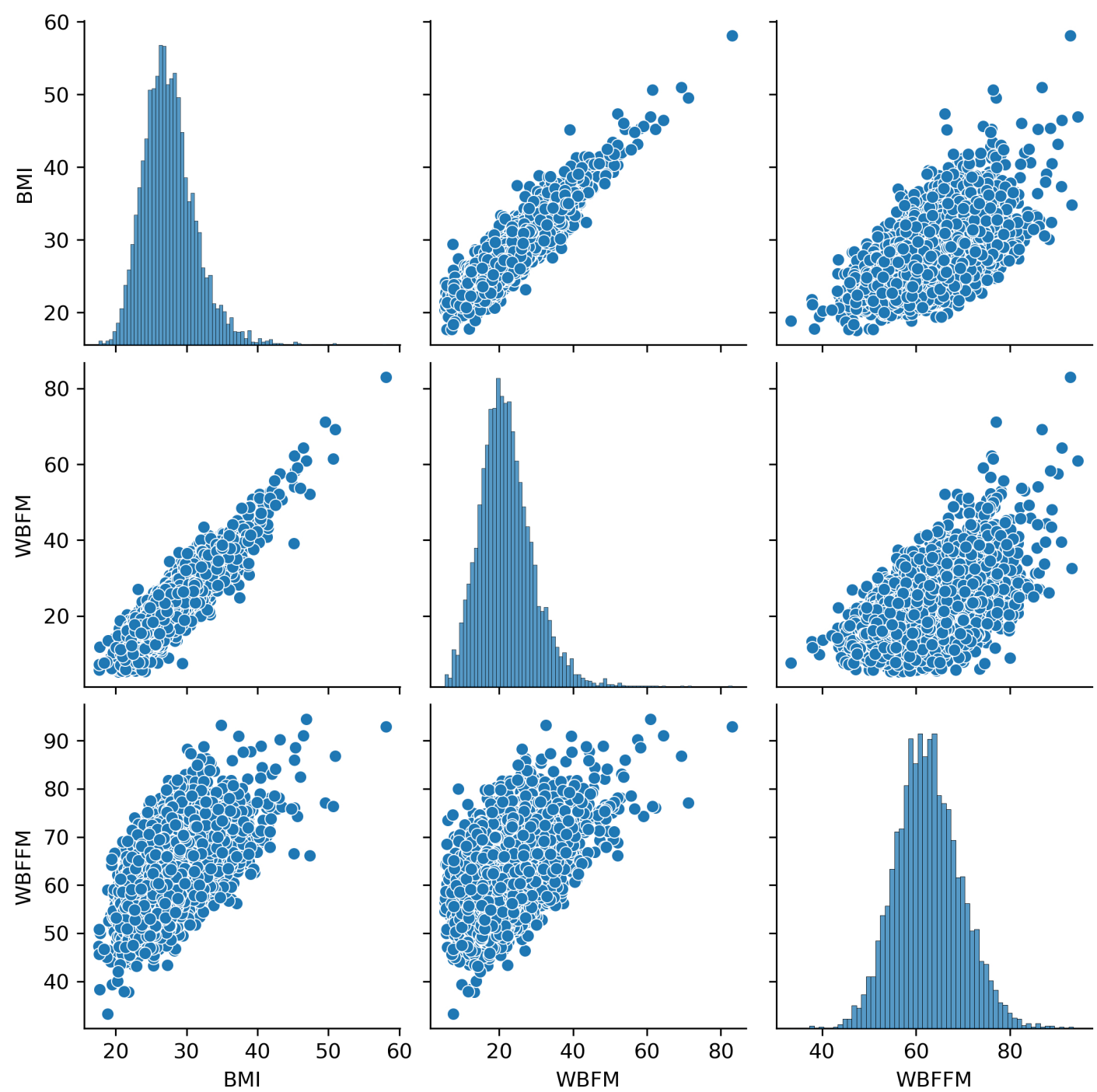

Supplement: Supplementary file 3 — Supplementary Figure 3. [file 41598_2024_54291_MOESM3_ESM.pdf]

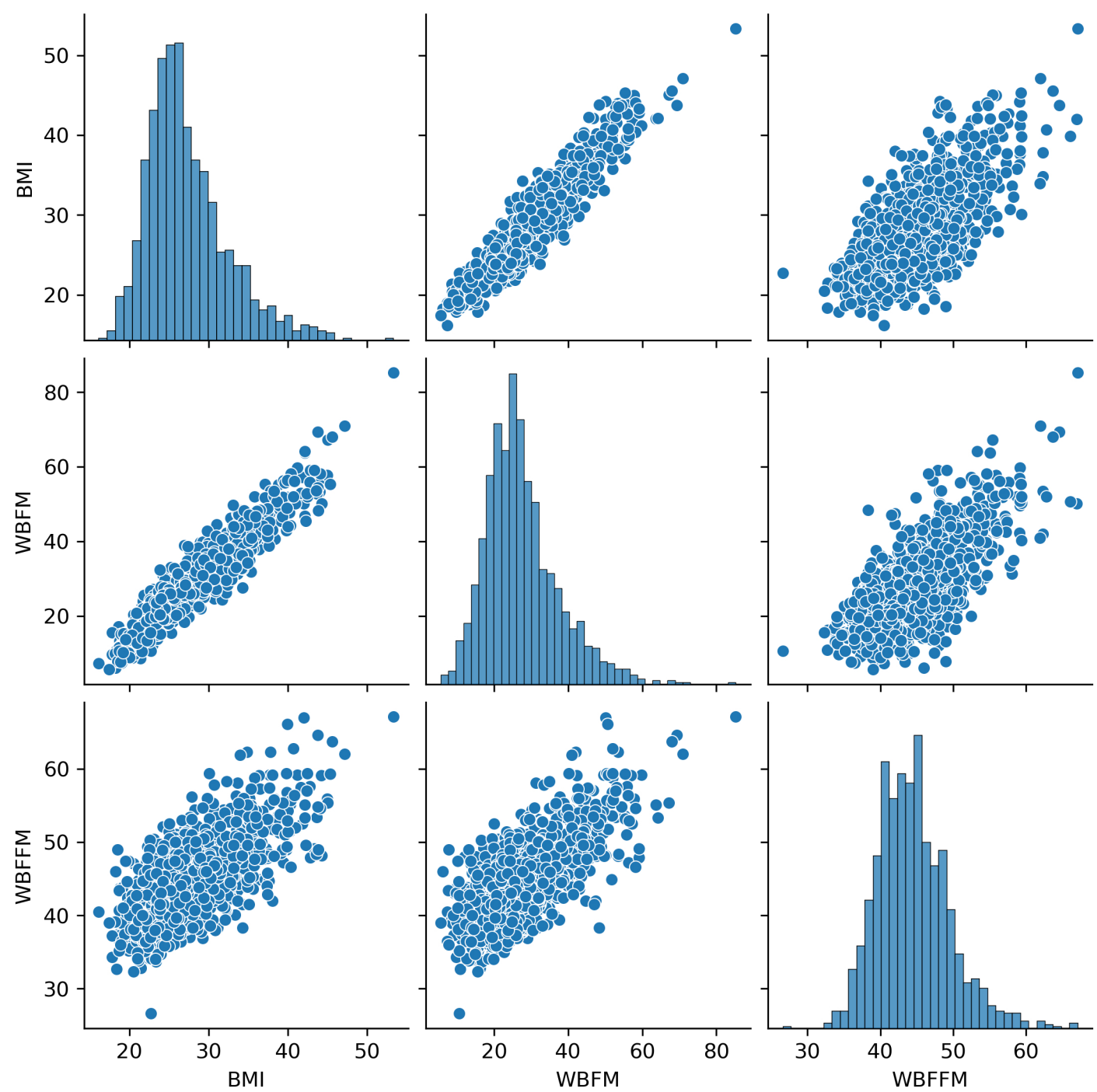

Supplement: Supplementary file 5 — Supplementary Figure 5. [file 41598_2024_54291_MOESM5_ESM.pdf]

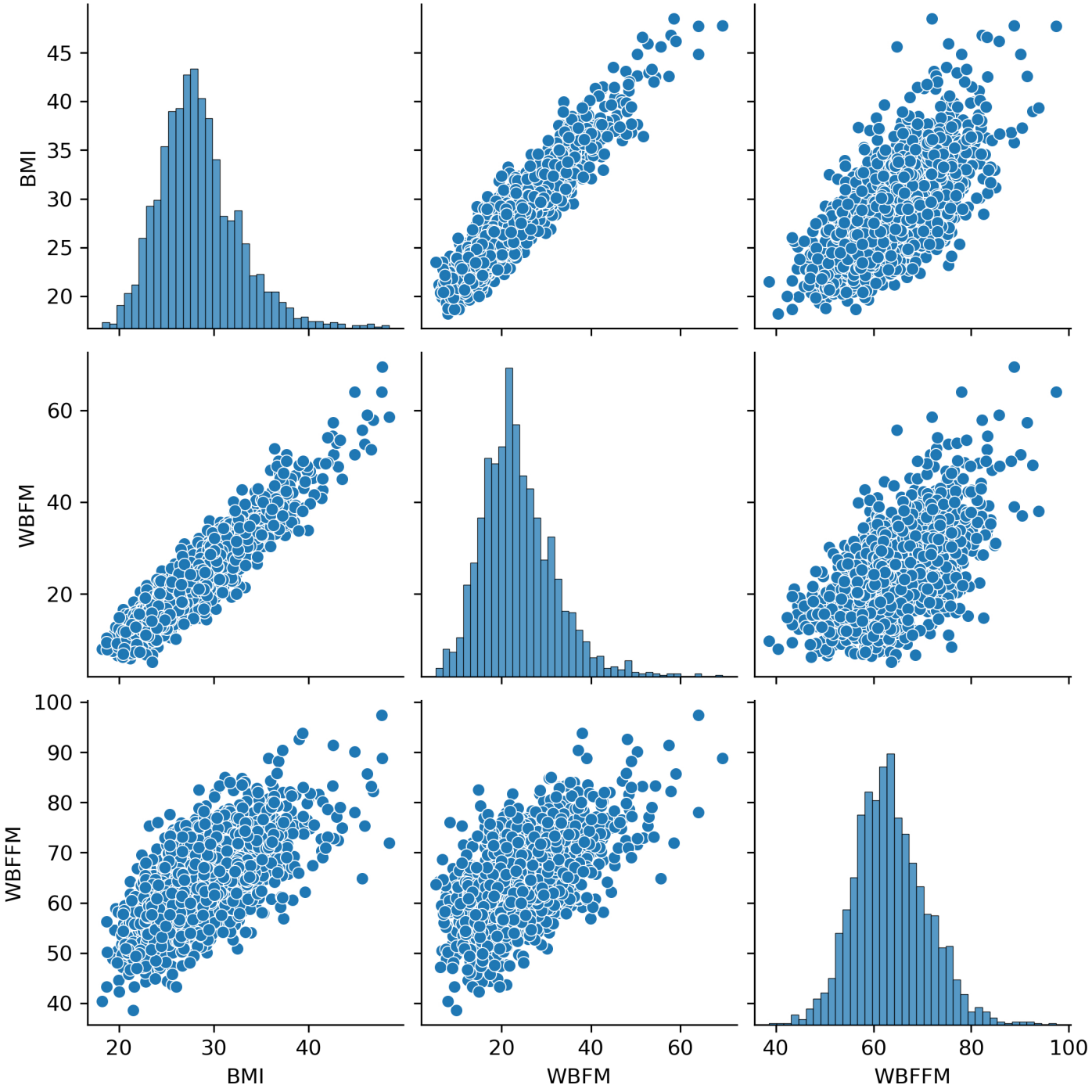

Supplement: Supplementary file 7 — Supplementary Figure 7. [file 41598_2024_54291_MOESM7_ESM.pdf]
