# Supplementary material for: New role of fat-free mass in cancer risk linked with genetic predisposition
Source: Sci Rep. 2024 Mar 27;14:7270. doi: 10.1038/s41598-024-54291-7 (PMC10973462; doi:10.1038/s41598-024-54291-7)
Supplement: Supplementary file 2 — Supplementary Figure 2. [file 41598_2024_54291_MOESM2_ESM.pdf]

a

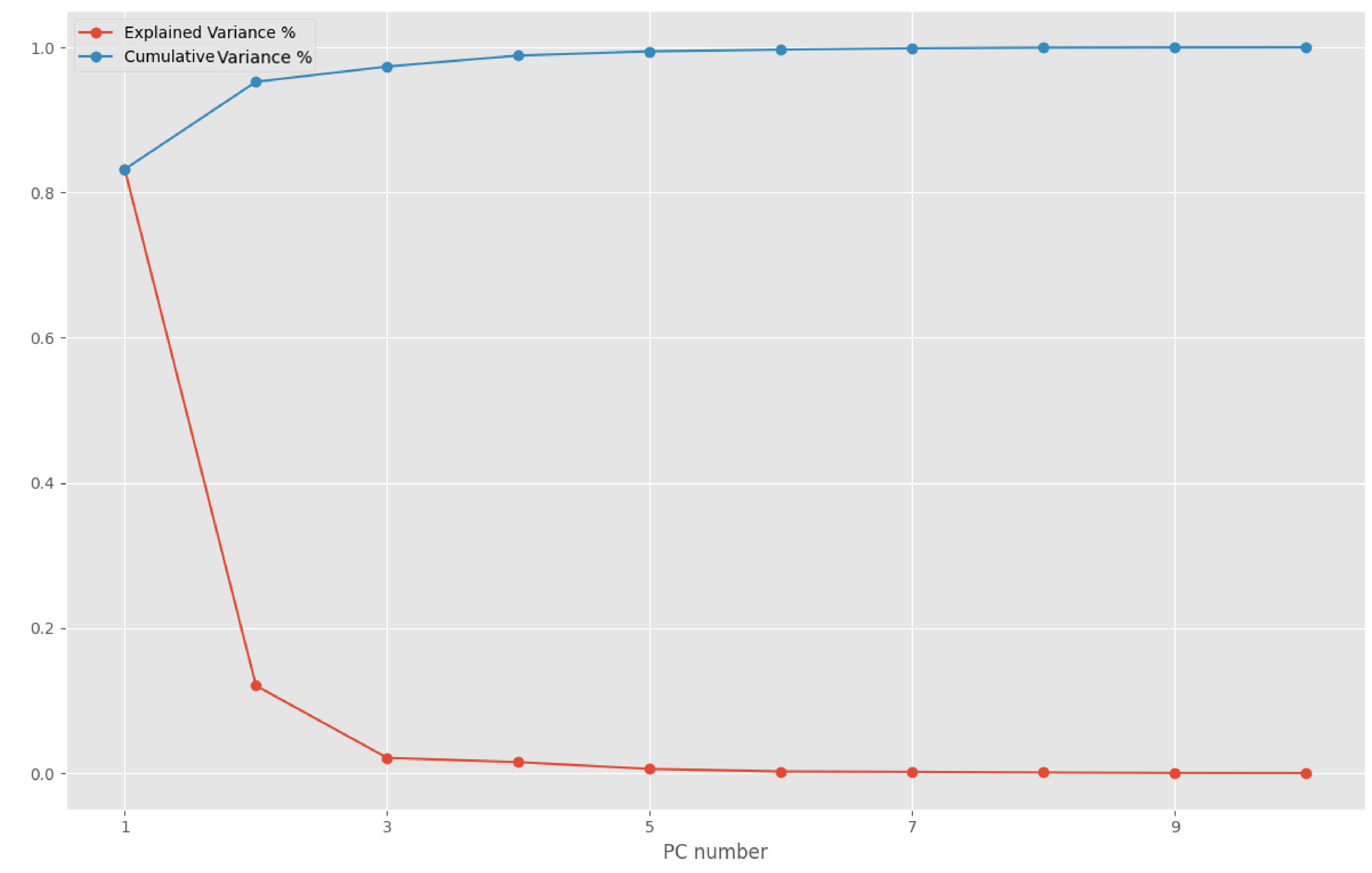

b

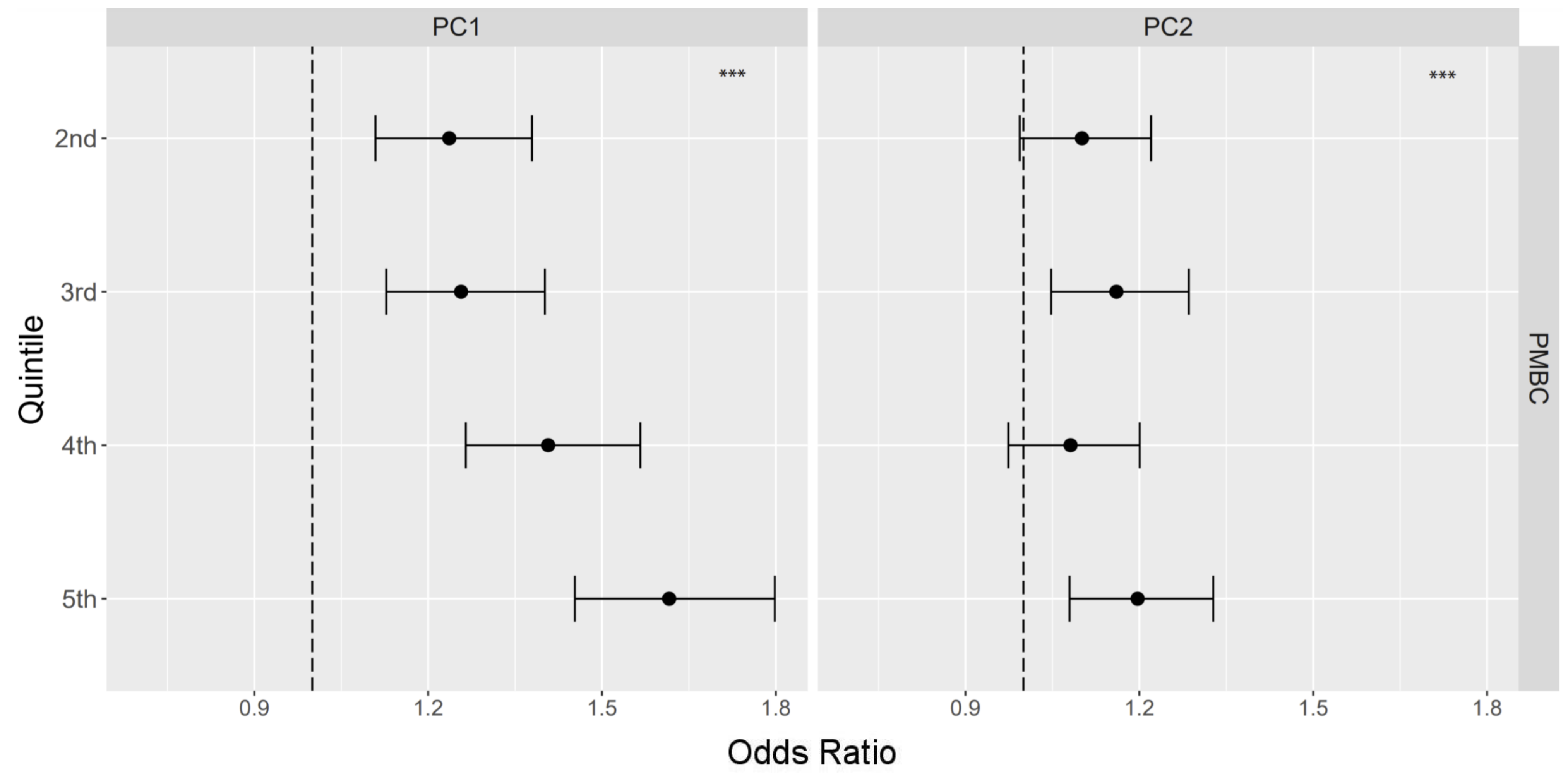

c

	PC1	PC2
**WBFM**	0.81	-0.28
**WBFFM**	0.32	0.78
**Leg fat mass (right)**	0.14	-0.01
**Leg fat-free mass (right)**	0.07	0.12
**Leg fat mass (left)**	0.14	-0.01
**Leg fat-free mass (left)**	0.07	0.11
**Arm fat mass (right)**	0.06	0.00
**Arm fat-free mass (right)**	0.02	0.04
**Arm fat mass (left)**	0.06	0.00
**Arm fat-free mass (left)**	0.02	0.04
**Trunk fat mass**	0.41	-0.25
**Trunk fat-free mass**	0.14	0.47
