# Supplementary material for: New role of fat-free mass in cancer risk linked with genetic predisposition
Source: Sci Rep. 2024 Mar 27;14:7270. doi: 10.1038/s41598-024-54291-7 (PMC10973462; doi:10.1038/s41598-024-54291-7)
Supplement: Supplementary file 4 — Supplementary Figure 4. [file 41598_2024_54291_MOESM4_ESM.pdf]

a

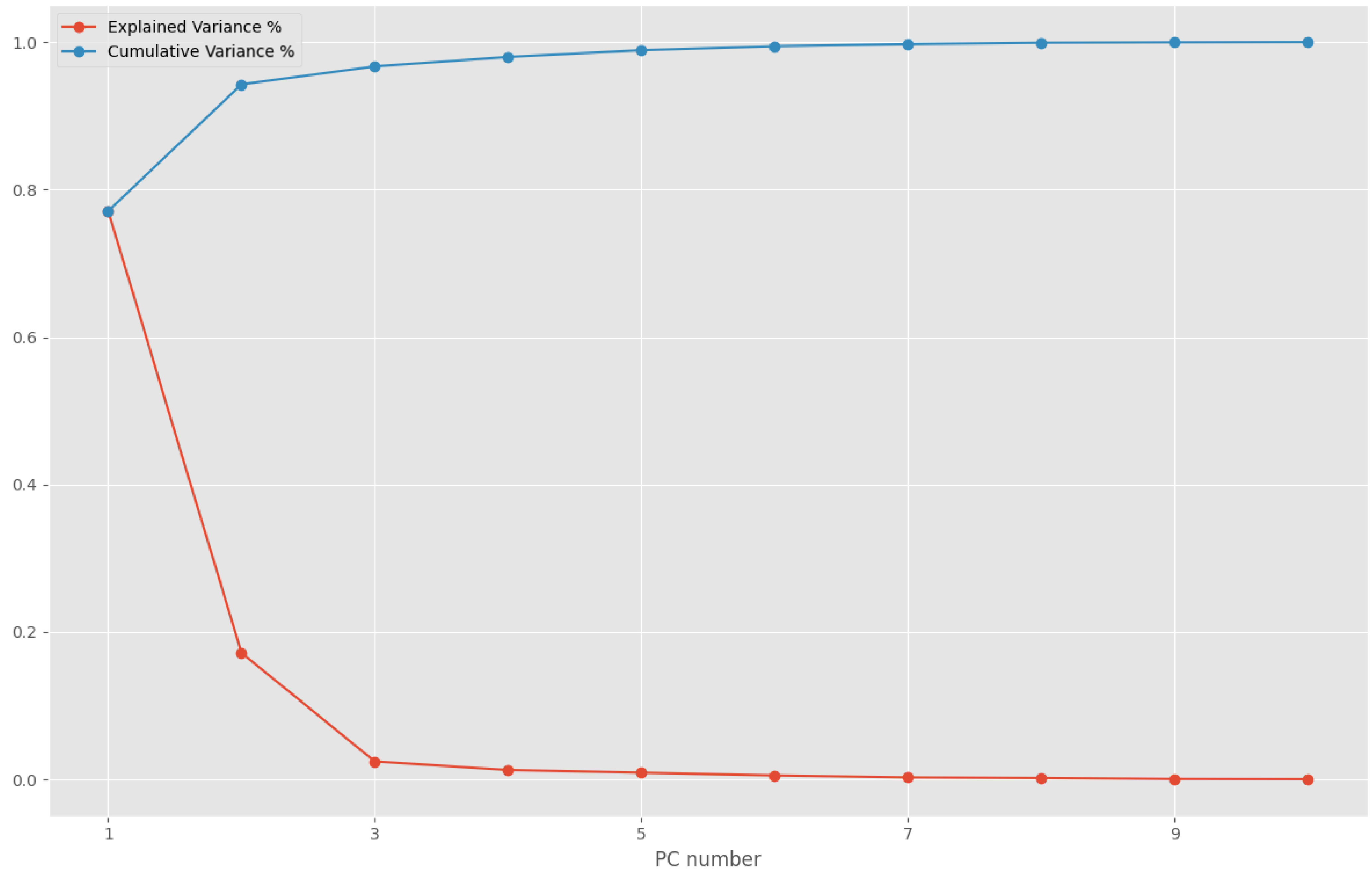


b

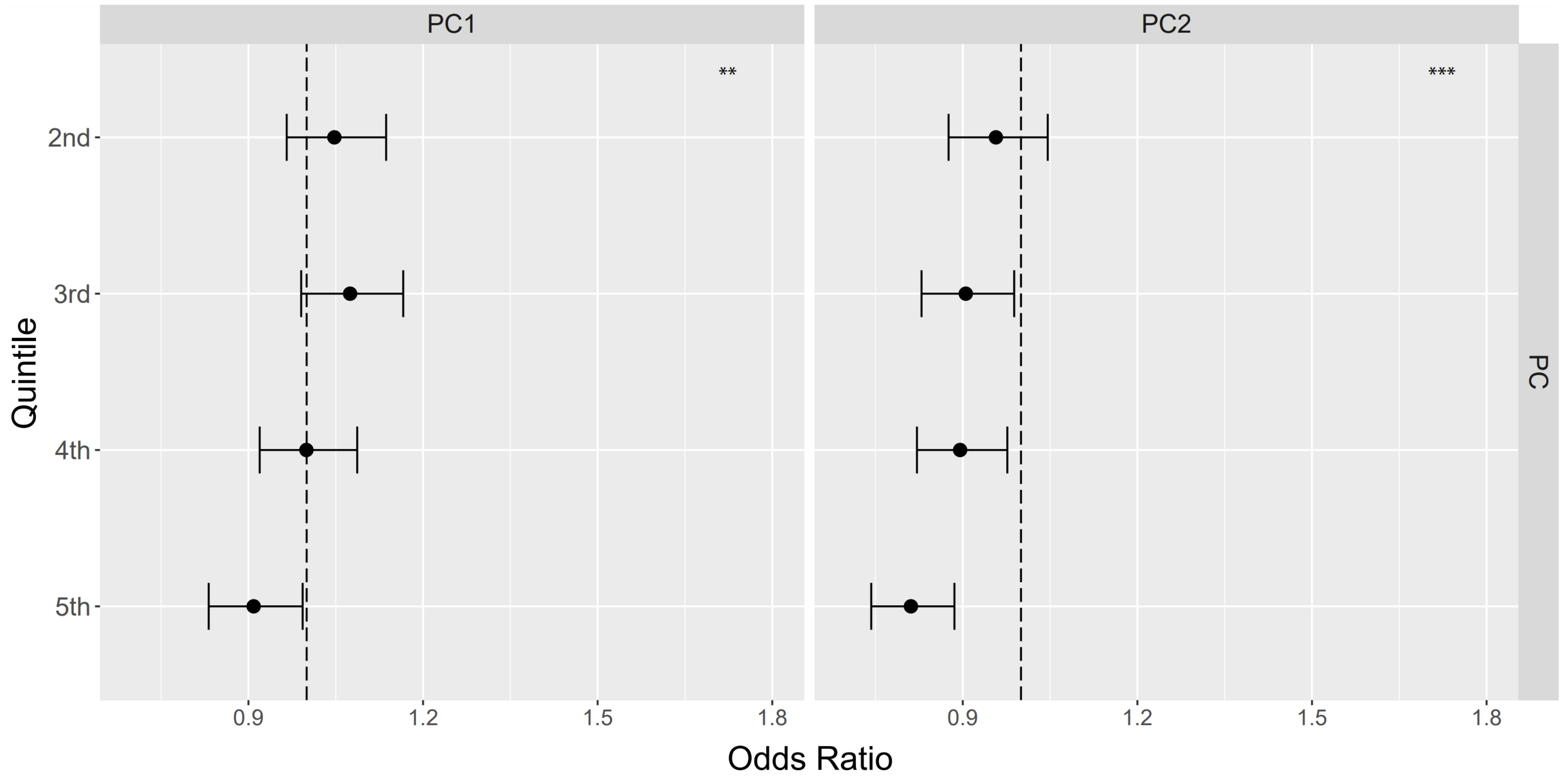


c

| | PC1 | PC2 |
|---|---|---|
| **WBFM** | 0.64 | 0.54 |
| **WBFFM** | 0.57 | -0.64 |
| **Leg fat mass (right)** | 0.09 | 0.08 |
| **Leg fat-free mass (right)** | 0.11 | -0.08 |
| **Leg fat mass (left)** | 0.09 | 0.07 |
| **Leg fat-free mass (left)** | 0.12 | -0.07 |
| **Arm fat mass (right)** | 0.04 | 0.02 |
| **Arm fat-free mass (right)** | 0.04 | -0.04 |
| **Arm fat mass (left)** | 0.04 | 0.02 |
| **Arm fat-free mass (left)** | 0.04 | -0.05 |
| **Trunk fat mass** | 0.38 | 0.34 |
| **Trunk fat-free mass** | 0.26 | -0.40 |
