# Supplementary material for: New role of fat-free mass in cancer risk linked with genetic predisposition
Source: Sci Rep. 2024 Mar 27;14:7270. doi: 10.1038/s41598-024-54291-7 (PMC10973462; doi:10.1038/s41598-024-54291-7)
Supplement: Supplementary file 8 — Supplementary Figure 8. [file 41598_2024_54291_MOESM8_ESM.pdf]

a

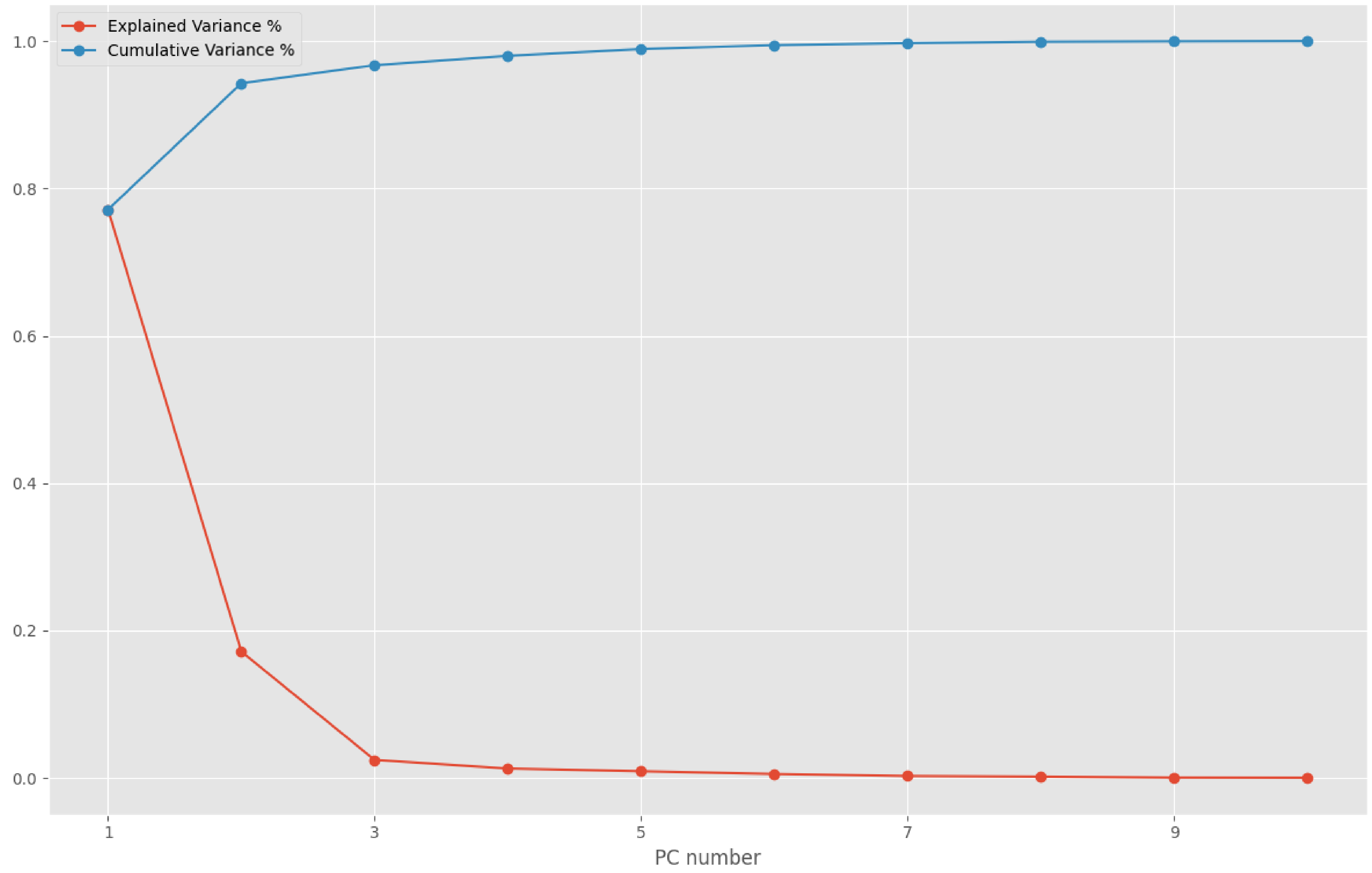

b

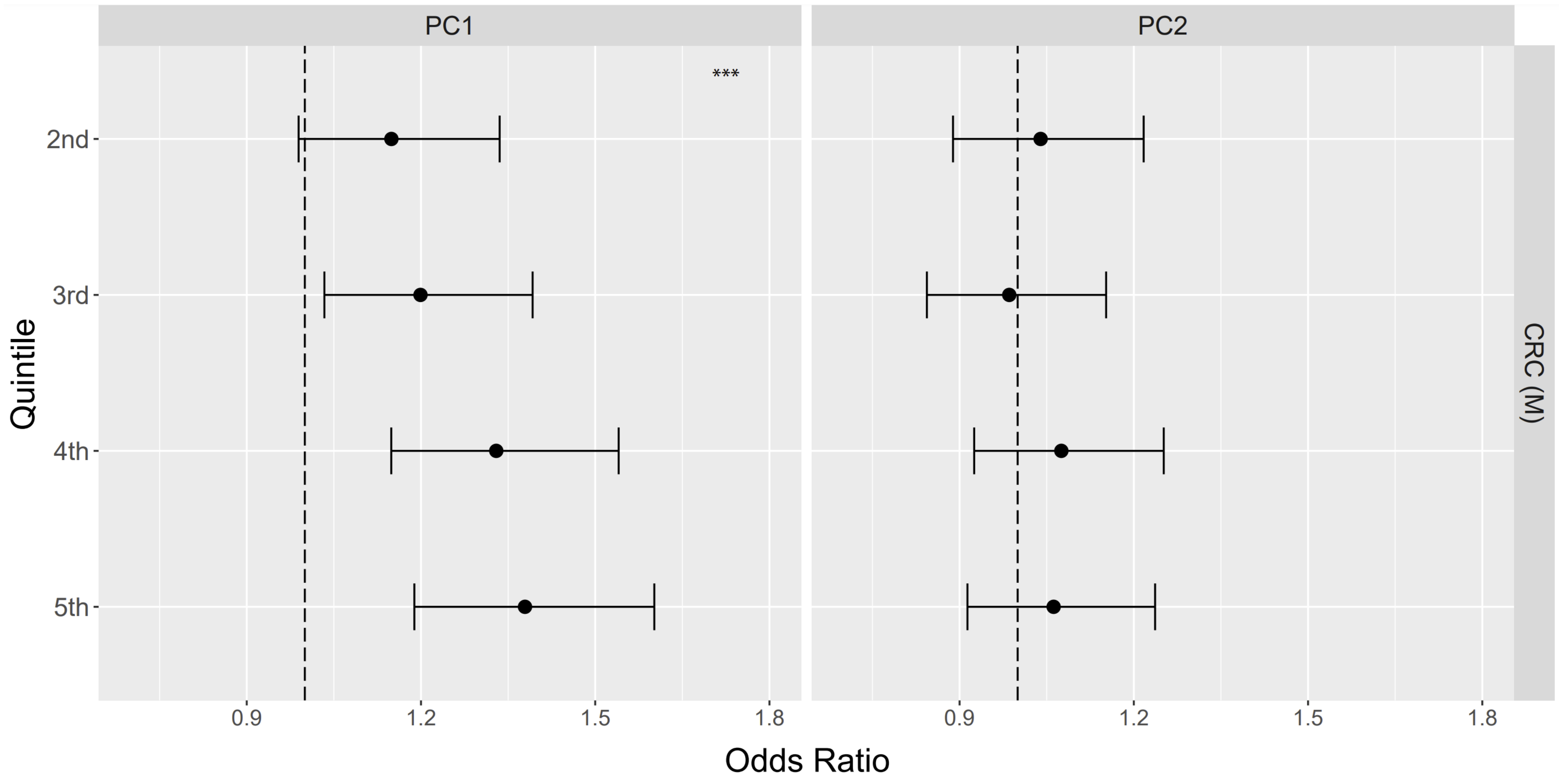

c

	PC1	PC2
**WBFM**	0.64	0.54
**WBFFM**	0.57	-0.64
**Leg fat mass (right)**	0.09	0.08
**Leg fat-free mass (right)**	0.11	-0.08
**Leg fat mass (left)**	0.09	0.07
**Leg fat-free mass (left)**	0.12	-0.07
**Arm fat mass (right)**	0.04	0.02
**Arm fat-free mass (right)**	0.04	-0.04
**Arm fat mass (left)**	0.04	0.02
**Arm fat-free mass (left)**	0.04	-0.05
**Trunk fat mass**	0.38	0.34
**Trunk fat-free mass**	0.26	-0.40
