# Supplementary material for: New role of fat-free mass in cancer risk linked with genetic predisposition
Source: Sci Rep. 2024 Mar 27;14:7270. doi: 10.1038/s41598-024-54291-7 (PMC10973462; doi:10.1038/s41598-024-54291-7)
Supplement: Supplementary file 9 — Supplementary Legends. [file 41598_2024_54291_MOESM9_ESM.docx]

## **Table S1:** *Number of cases of each cancer type within the quintiles of each anthropometric measure.* The number of cases are displayed for post-menopausal breast cancer (a), prostate cancer (b), colorectal cancer in women (c) and colorectal cancer in men (d) which are used in the multivariable regression analyses.

## **Table S2:** *Quintile cut-off points in the multivariable regression analyses*

## **Table S3:** *Spearman’s rank correlation coefficients of BMI, WBFM, WBFFM and Standing Height.* These are the values for the post-menopausal breast cancer analysis cohort (a), the prostate cancer analysis cohort (b), the female colorectal cancer analysis cohort (c) and the male colorectal cancer analysis cohort (d). P values for all correlations are <0.001.

## **Figure S1:** *Anthropometric traits in the post-menopausal breast cancer cohort.* a) Data distribution of BMI, WBFM and WBFFM. In the dot plots, the axes correspond to units of measure BMI [kg/m2], WBFM [kg], WBFFM [kg] and red dots = cancer cases. Univariate plots (on the diagonal) represent the distribution of measures within the population with the horizontal axis = the anthropometric measure with aforementioned units and vertical axis as frequency count. n = 131,546 women (3,952 cases and 127,594 controls).

## **Figure S2:** *Principal component analysis of post-menopausal breast cancer risk.* Panel A: normalised percentage of variance explained by each principal component. Panel B: risk of post-menopausal breast cancer in the different quintiles of principal component 1 (PC1) and principal component (PC2) compared to the lowest quintile (OR with 95% confidence interval). PMBC = post-menopasual breast cancer, *** = p trend <0.001. Panel C: loading values for anthropometric measures in PC1 and PC2. Cohort as in Fig. S1.

## **Figure S3:** *Anthropometric traits in prostate cancer cohort.* a) Data distribution of BMI, WBFM and WBFFM. In the dot plots, the axes correspond to units of measure BMI [kg/m2], WBFM [kg], WBFFM [kg] and red dots = cancer cases. Univariate plots (on the diagonal) represent the distribution of measures within the population with the horizontal axis = the anthropometric measure with aforementioned units and vertical axis as frequency count. n = 191,434 men (5,982 cases and 185,452 controls).

## **Figure S4:** *Principal component analysis of prostate cancer risk.* Panel A: normalised percentage of variance explained by each principal component. Panel B: risk of prostate cancer in the different quintiles of principal component 1 (PC1) and principal component (PC2) compared to the lowest quintile (OR with 95% confidence interval). PC = prostate cancer, ** = p trend <0.01, *** = p trend <0.001. Panel C: loading values for anthropometric measures in PC1 and PC2. Cohort as in Fig. S3.

## **Figure S5:** *Anthropometric traits in women in colorectal cancer cohort.* a) Data distribution of BMI, WBFM and WBFFM. In the dot plots, the axes correspond to units of measure BMI [kg/m2], WBFM [kg], WBFFM [kg] and red dots = cancer cases. Univariate plots (on the diagonal) represent the distribution of measures within the population with the horizontal axis = the anthropometric measure with aforementioned units and vertical axis as frequency count. n = 218,954 women (1,424 cases and 217,530 controls).

## **Figure S6:** *Principal component analysis of colorectal cancer risk in women.* Panel A: normalised percentage of variance explained by each principal component. Panel B: risk of colorectal cancer in the different quintiles of principal component 1 (PC1) and principal component (PC2) compared to the lowest quintile (OR with 95% confidence interval). CRC (F) = colorectal cancer in women, *** = p trend <0.001. Panel C: loading values for anthropometric measures in PC1 and PC2. Cohort as in Fig. S5.

## **Figure S7:** *Anthropometric traits in men in colorectal cancer cohort.* a) Data distribution of BMI, WBFM and WBFFM. In the dot plots, the axes correspond to units of measure BMI [kg/m2], WBFM [kg], WBFFM [kg] and red dots = cancer cases. Univariate plots (on the diagonal) represent the distribution of measures within the population with the horizontal axis = the anthropometric measure with aforementioned units and vertical axis as frequency. n = 187,439 men (1,996 cases and 185,443 controls).

## **Figure S8:** *Principal component analysis of colorectal cancer risk in men.* Panel A: normalised percentage of variance explained by each principal component. Panel B: risk of colorectal cancer in the different quintiles of principal component 1 (PC1) and principal component (PC2) compared to the lowest quintile (OR with 95% confidence interval). CRC (M) = colorectal cancer in men, *** = p trend <0.001. Panel C: loading values for anthropometric measures in PC1 and PC2. Cohort as in Fig. S7.
