# Supplementary material for: New role of fat-free mass in cancer risk linked with genetic predisposition
Source: Sci Rep. 2024 Mar 27;14:7270. doi: 10.1038/s41598-024-54291-7 (PMC10973462; doi:10.1038/s41598-024-54291-7)
Supplement: Supplementary file 10 — Supplementary Table 1. [file 41598_2024_54291_MOESM10_ESM.pdf]

a

	BMI		WBFM		WBFFM	
	PMBC Cases	***No PMBC***	***PMBC Cases***	***No PMBC***	***PMBC Cases***	***No PMBC***
**Quintile 1**	650	25673	621	25862	646	26389
**Quintile 2**	758	25551	729	25594	738	24865
**Quintile 3**	780	25539	813	25492	787	26104
**Quintile 4**	842	25449	843	25333	840	25368
**Quintile 5**	922	25382	946	25313	941	24868

b

	BMI		WBFM		WBFFM	
	PC Cases	***No PC***	***PC Cases***	***No PC***	***PC Cases***	***No PC***
**Quintile 1**	1186	37106	1128	37894	1343	37487
**Quintile 2**	1269	37014	1207	36398	1378	36902
**Quintile 3**	1273	37016	1327	37459	1263	37041
**Quintile 4**	1241	37042	1243	36525	1124	37242
**Quintile 5**	1013	37274	1077	37176	874	36780

c

	BMI		WBFM		WBFFM	
	CRC (F) Cases	***No CRC (F)***	***CRC (F) Cases***	***No CRC (F)***	***CRC (F) Cases***	***No CRC (F)***
**Quintile 1**	234	43558	235	43647	298	44084
**Quintile 2**	287	43528	277	44277	270	43111
**Quintile 3**	326	43446	320	42776	292	44747
**Quintile 4**	280	43529	293	43625	290	43057
**Quintile 5**	297	43469	299	43205	274	42531

d

	BMI		WBFM		WBFFM	
	CRC (M) Cases	***No CRC (M)***	***CRC (M) Cases***	***No CRC (M)***	***CRC (M) Cases***	***No CRC (M)***
**Quintile 1**	319	37197	300	37892	409	37486
**Quintile 2**	338	37133	341	37460	419	36903
**Quintile 3**	431	37050	423	36395	428	37032
**Quintile 4**	441	37043	434	37099	370	37242
**Quintile 5**	467	37020	498	36597	370	36780
