# Supplementary material for: New role of fat-free mass in cancer risk linked with genetic predisposition
Source: Sci Rep. 2024 Mar 27;14:7270. doi: 10.1038/s41598-024-54291-7 (PMC10973462; doi:10.1038/s41598-024-54291-7)
Supplement: Supplementary file 11 — Supplementary Table 2. [file 41598_2024_54291_MOESM11_ESM.pdf]

a

	BMI	WBFM	WBFFM
**Quintile 1**	15.0 - 23.0	5.0 - 19.1	26.2 - 40.0
**Quintile 2**	23.0 - 25.2	19.1 - 23.4	40.0 - 42.3
**Quintile 3**	25.2 - 27.4	23.4 - 27.8	42.3 - 44.6
**Quintile 4**	27.4 - 30.7	27.8 - 34.0	44.6 - 47.6
**Quintile 5**	30.7 - 68.1	34.0 - 108.4	47.6 - 82.7

b

	BMI	WBFM	WBFFM
**Quintile 1**	15.0- 24.5	5.0 - 15.7	33.0 - 57.3
**Quintile 2**	24.5 - 26.4	15.7 - 19.4	57.3 - 61.4
**Quintile 3**	26.4 - 28.3	19.4 - 23.1	61.4 - 65.2
**Quintile 4**	28.3 - 30.8	23.1 - 28.0	65.2 - 70.0
**Quintile 5**	30.8 - 68.4	28.0 - 108.1	70.0 - 100.0

c

	BMI	WBFM	WBFFM
**Quintile 1**	14.5 - 22.9	4.999 - 18.6	26.2 - 40.4
**Quintile 2**	22.9 - 25.0	18.6 - 23.1	40.4 - 42.8
**Quintile 3**	25.0 - 27.3	23.1 - 27.6	42.8 - 45.2
**Quintile 4**	27.3 - 30.8	27.6 - 34.2	45.2 - 48.3
**Quintile 5**	30.8 - 68.1	34.2 - 109.8	48.3 - 84.9

d

	BMI	WBFM	WBFFM
**Quintile 1**	15.0 - 24.5	5.0 - 15.7	33.0 - 57.3
**Quintile 2**	24.5 - 26.4	15.7 - 19.5	57.3 - 61.4
**Quintile 3**	26.4 - 28.3	19.5 - 23.1	61.4 - 65.2
**Quintile 4**	28.3 - 30.9	23.1 - 28.1	65.2 - 70.0
**Quintile 5**	30.9 - 68.4	28.1 - 108.1	70.0 - 100.0
