# Supplementary material for: New role of fat-free mass in cancer risk linked with genetic predisposition
Source: Sci Rep. 2024 Mar 27;14:7270. doi: 10.1038/s41598-024-54291-7 (PMC10973462; doi:10.1038/s41598-024-54291-7)
Supplement: Supplementary file 12 — Supplementary Table 3. [file 41598_2024_54291_MOESM12_ESM.pdf]

a

	BMI	WBFM	WBFFM
**BMI**	1	0.92	0.62
**WBFM**	0.92	1	0.64
**WBFFM**	0.62	0.64	1

b

	BMI	WBFM	WBFFM
**BMI**	1	0.89	0.61
**WBFM**	0.89	1	0.53
**WBFFM**	0.61	0.53	1

c

	**BMI**	**WBFM**	**WBFFM**
**BMI**	1	0.93	0.61
**WBFM**	0.93	1	0.63
**WBFFM**	0.61	0.63	1

d

	**BMI**	**WBFM**	**WBFFM**
**BMI**	1	0.90	0.61
**WBFM**	0.90	1	0.53
**WBFFM**	0.61	0.53	1
